# Comparing the incomparable: Population densities and first responder groups through alternate statistics

**DOI:** 10.1016/j.resplu.2025.101130

**Published:** 2025-10-15

**Authors:** Petra Szvath, Thijs Gloger, Cecilie Langkilde Lauesen, Barry Murphy-Jones, Sebastian Schnaubelt

**Affiliations:** a Department of Anesthesiology and Intensive Therapy, Semmelweis University, Budapest, Hungary; b Scientific Committee of Hungarian Resuscitation Council, Hungary; c Witte Kruis Ambulance Trust, Zeeland, Middelburg, Netherlands; d Department of Medicine, Randers Regional Hospital, Randers, Denmark; e Research Center for Emergency Medicine, Aarhus University Hospital, Aarhus, Denmark; f London Ambulance Service NHS Trust and University of Hertfordshire, United Kingdom; g Department of Emergency Medicine, Medical University of Vienna and Emergency Medical Service, Vienna, Austria

**Keywords:** Out-of-hospital cardiac arrest, Emergency medical services, Fire and rescue services, Volunteer community responders, Response times, Journal club, Study review

**Figure:**
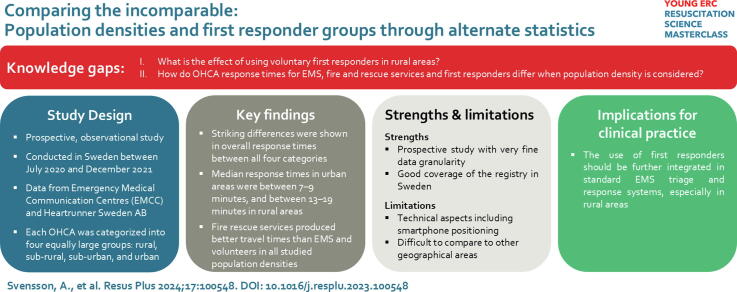

## Introduction

This article is based on a Journal Club presented in the Young ERC Resuscitation Science Masterclass^1^ in which the study “Response times in rural areas for emergency medical services, fire and rescue services and volunteer community responders during out-of-hospital cardiac arrests”^2^ was presented.

## Which knowledge gap does this study try to fill out?

Survival after out-of-hospital cardiac arrest (OHCA) depends on immediate initiation of cardiopulmonary resuscitation (CPR).^3^, ^4^ Thus, short Emergency Medical Service (EMS) response times are crucial.^5^, ^6^, ^7^, ^8^ To decrease time to CPR, fire and rescue services as well as volunteer community responders are utilised. However, volunteer community responders are mostly alerted in urban areas even though the most prolonged EMS response times are seen in rural areas.^9^, ^10^, ^11^, ^12^

The knowledge gaps investigated in the study are: (1) What is the effect of using volunteer community responders in rural areas?, and (2) How do OHCA response times for EMS, fire and rescue, and community responders differ when population density is taken into consideration?

## What was the design of the study?

This is a prospective, observational study conducted in a region in southern Sweden between July 2020 and December 2021.

Data were collected from Emergency Medical Communication Centres (EMCC) and Heartrunner Sweden AB which is the system behind a smartphone application alarming volunteer community responders.^4^ Data included event times for EMS, fire and rescue services and community responders. The process time (from EMCC answering the emergency call to EMCC dispatching EMS + fire and rescue services and alerting community responders), turnout time (from dispatch to departure), travel time (from departure to arrival on scene), and response time (the sum of process time, turnout time and travel time) were analysed for each instance.

**Fig. 1:**
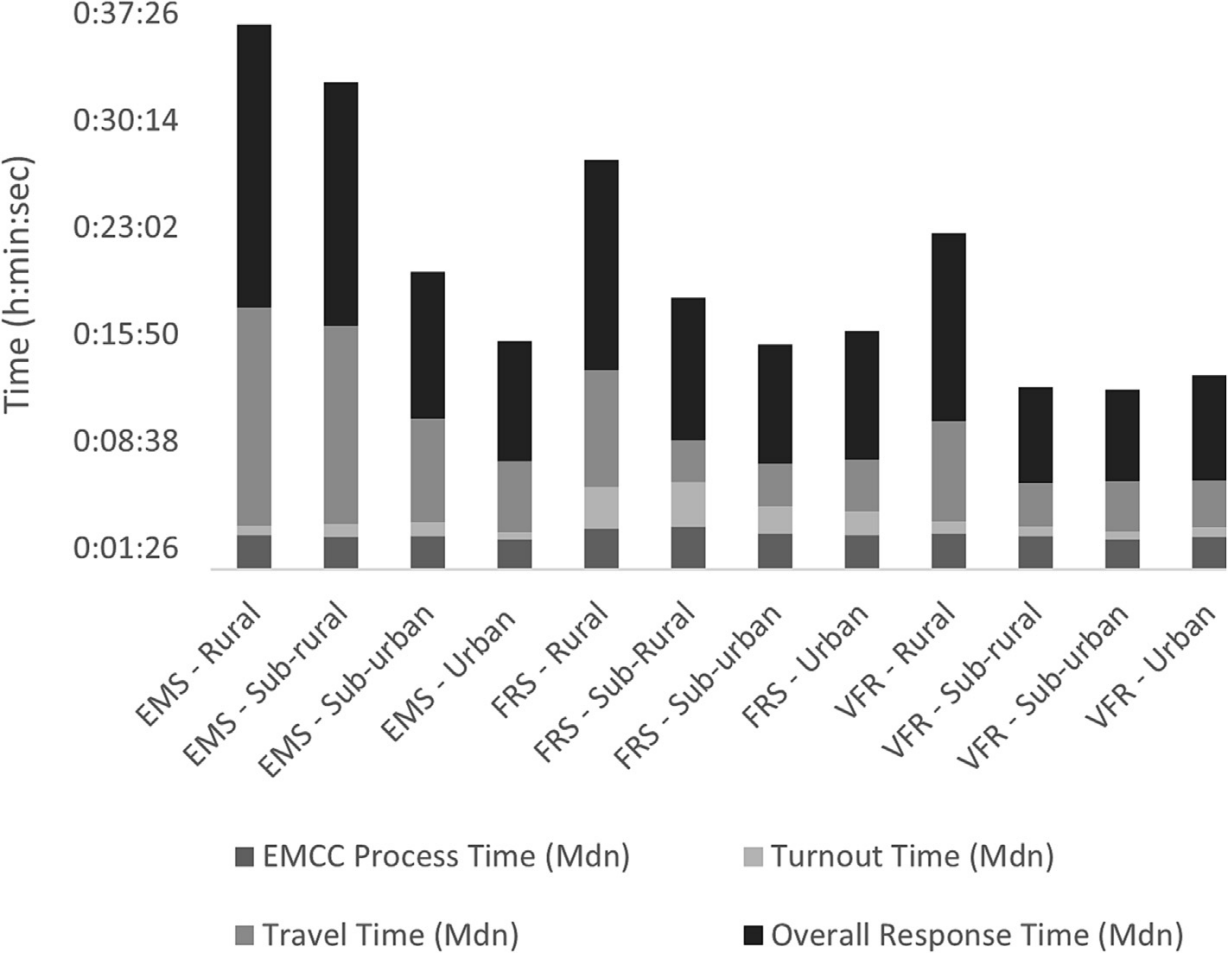
Different times (median values) depending on service and population density. EMS: Emergency Medical Services, FRS: Fire and Rescue Services, VFR: Volunteer First Responders. (Source of image: made by the authors of this article, modified after the published data of the cited article^2^)

In 2020, the population density of the region was 24/km^2^, with a range of municipalities from 8.1 to 57.0 inhabitants/km^2^. Each OHCA was ranked from the smallest to the largest population density and categorized into four groups: rural, sub-rural, sub-urban, and urban (Fig. 1).

## What were the key findings of the study?

Regarding the overall response times, the differences between the rural, sub-rural, sub-urban, and urban areas are striking, irrespective of the service unit. While the median response times in urban areas was between 7 and 9 minutes, they were between 13 and 19 minutes in rural areas, meaning that none of the services were able to arrive in time to achieve the goal set by the Swedish Resuscitation Council of OHCA which is to perform defibrillation within five minutes after cardiac arrest.^13^

The travel times show the most salient differences; the delay of EMS compared to fire and rescue services and community responders is clearly visible, especially in non-urban areas. In contrast, in urban areas, EMS and fire rescue services had similar median response times (around 8 minutes), differing by only 30 seconds.

Fire and rescue services produced similar but somewhat longer travel times than community responders in all studied population densities, which – compared to EMS – still leads to a prominent time gain in rural areas. For fire and rescue services, longer turnout times can be observed; the discrepancy was explained by the differences in the alerting of full-time and part-time firefighters.

Community responders had significantly shorter response times compared to EMS and fire and rescue services. The median response times by community responders were shorter by nearly 3 minutes compared to the fire rescue services and over 5 minutes compared to EMS. Of particular note, were response times in the sub-rural areas where response times by community responders were shorter by 7 and 10 minutes compared to fire rescue services and EMS, respectively. Nevertheless, in more than half of the cases (54 %) EMS and/or Fire and rescue services were first on scene. It must be acknowledged that only a small proportion (19 %) of the assignments were accepted by community responders, despite a growing total number of alerts.

## Are there any important methodological considerations to learn from the study?

Because of the substantial differences in population density in the studied area, direct comparisons between actual geographical clusters were not feasible. To conquer this statistical challenge, the study authors decided to organize data into four categories, based on geodata and population density per km^2^. OHCA cases were divided into four clusters (rural, sub-rural, sub-urban, urban), with each cluster containing equally 71 cases. This methodological approach leads to some concerns about the reliability of the results. The impact of population density on the different response times could have been more pronounced if the four categories were based on actual, probably uneven distribution of the cases. The original article included a map signalling every alert that shows the diversity of cases near cities. This raises the question; is it the population density of the given area or the geographical distance to the closest responder that matters in a case of an emergency call for resuscitation? Though the results of the study show unambiguous evidence of the differences in the rural, sub-rural, sub-urban and urban categories, the adequacy of these categories is questionable.

## What are the most important strengths and limitations of the study?

One of the main strengths of the study is the authors’ resourcefulness in using an unconventional statistical methodology to compare different datasets. Furthermore, it is a prospective study with fine data granularity, especially regarding the time stamps. Offering detail in this field, the study underlines the importance of non-EMS responders in the treatment of OHCA. Another strength of the study is the great coverage of the registry in Sweden.

Limitations are found in technical aspects including estimated arrival times of community responders due to smartphone positioning and some cases of fire and rescue services having not confirmed their arrival. Some important features of this study are the relatively low overall population density and quite specific characteristics of the researched area in Sweden. Given the fact that even the “urban” / high population density cluster has a population density of >57/km^2^ that is below the mean population density in 36 other European countries, the results of this study are difficult to compare to other countries. Thus, when the applicability of these results comes to question, we suggest interpreting the population density categories accordingly – these are essentially typical to rural and very rural areas in most European countries. Population density in this study was calculated within a radius of 1800 m around the arrest location. While this standardized measure is crucial in the evaluation of the specific data of this study, the choice of radius is somewhat arbitrary. It remains uncertain which spatial scale would be most relevant when assessing the influence of population density on cardiac arrest outcomes. Also, no details are mentioned about the level of training of non-EMS responders. There is lack of data on patient characteristics, demographic data, AED/defibrillator availability, or number of attempted resuscitations. Moreover, this study was partly carried out during the COVID-19 pandemic, which could have influenced community responders’ willingness and availability to respond.^14^

From a strictly communicative point of view, graphical presentations of the data are lacking. Charts or graphics of any kind might have assisted the reader in grasping the most important tendencies.

## How will the results affect your clinical practice? What do you see as the next steps in research?

The use of volunteer community responders forms an important part of the chain of survival.^15^ The study shows that even in urban areas community responders can arrive on scene before EMS and fire and rescue services. Importantly, in rural areas where EMS response times are prolonged, community responders can arrive significantly quicker. The use of community responders should be further integrated in standard EMS triage and response systems, especially in rural areas, as the use of volunteer community responders with AEDs has been shown to double survival to discharge of witnessed OHCA.^16^

Further research should consider the reasons for low numbers of voluntary responders and non-acceptance of tasks. The diversity of data according to different geographical and population density categories is to be considered in the planning of future studies assessing the differences of CPR in urban and rural environment.

## CRediT authorship contribution statement

**Petra Szvath:** Writing – review & editing, Writing – original draft, Visualization, Project administration, Methodology, Investigation, Formal analysis, Data curation, Conceptualization. **Thijs Gloger:** Writing – review & editing, Writing – original draft, Methodology, Investigation, Conceptualization. **Cecilie Langkilde Lauesen:** Writing – review & editing, Writing – original draft, Project administration, Methodology, Investigation, Formal analysis, Conceptualization. **Barry Murphy-Jones:** Writing – review & editing, Writing – original draft, Methodology, Investigation, Conceptualization. **Sebastian Schnaubelt:** Writing – review & editing, Supervision, Funding acquisition, Conceptualization.

## Declaration of competing interest

The authors declare that they have no known competing financial interests or personal relationships that could have appeared to influence the work reported in this paper.
